# Evaluation of Spatial Gas Temperature and Water Vapor Inhomogeneities in TDLAS in Circular Multipass Absorption Cells Used for the Analysis of Dynamic Tube Flows

**DOI:** 10.3390/s23094345

**Published:** 2023-04-27

**Authors:** Felix Witt, Henning Bohlius, Volker Ebert

**Affiliations:** Physikalisch-Technische Bundesanstalt, Bundesallee 100, 38116 Braunschweig, Germany; felix.witt@ptb.de (F.W.); henning.bohlius@ptb.de (H.B.)

**Keywords:** spatial inhomogeneities, laser spectroscopy, tunable diode laser absorption spectroscopy, dTDLAS, circular multipass absorption cell, CMPAC, water vapor

## Abstract

The use of optical circular multipass absorption cells (CMPAC) in an open-path configuration enables the sampling free analysis of cylindrical gas flows with high temporal resolution and only minimal disturbances to the sample gas in the pipe. Combined with their robust unibody design, CMPACs are a good option for many applications in atmospheric research and industrial process monitoring. When deployed in an open-path configuration, the effects of inhomogeneities in the gas temperature and composition have to be evaluated to ensure that the resulting measurement error is acceptable for a given application. Such an evaluation needs to consider the deviations caused by spectroscopic effects, e.g., nonlinear effects of temperature variations on the intensity of the spectral line, as well as the interaction of the temperature and concentration field with the characteristic laser beam pattern of the CMPAC. In this work we demonstrate this novel combined evaluation approach for the CMPAC used as part of the tunable diode laser absorption spectroscopy (TDLAS) reference hygrometer in PTB’s *dynH_2_O* setup for the characterization of the dynamic response behavior of hygrometers. For this, we measured spatially resolved, 2D temperature and H_2_O concentration distributions, and combined them with spatially resolved simulated spectra to evaluate the inhomogeneity effects on the line area of the used H_2_O spectral line at 7299.43 cm^−1^. Our results indicate that for *dynH_2_O*, the deviations caused by the interaction between large concentration heterogeneities and the characteristic sampling of the beam pattern of the CMPAC are three orders of magnitude larger than deviations caused by small temperature heterogeneity induced spectroscopic effects. We also deduce that the assumption that the “path-integrated” H_2_O concentration derived with the open-path CMPAC setup represents an accurate H_2_O area average in the flow section covered by the CMPAC in fact shows significant differences of up to 16% and hence does not hold true when large H_2_O concentration gradients are present.

## 1. Introduction

Robust, optical multipass cells are frequently used for high-speed atmospheric water vapor measurements, especially on airborne carriers or even for flux measurements using the eddy covariance method [1,2,3,4]. Optical circular multipass absorption cells (CMPAC) [5,6], particularly when converted in an open-path version (opCMPAC) [3], offer—in contrast to White or Herriott cell configurations [7,8]—several benefits for sampling free, high-speed analyses of cylindrical gas flows with highly dynamic concentration changes. Tubular gas sampling free flows offer advantages during airborne atmospheric measurements as they optimize the throughput and hence response time, while minimizing sample surface contact and sample falsification by adsorption [3]. Cylindrical tube flows, however, are also ubiquitous in industry, hence in process monitoring and industrial process control, e.g., in power plants [9]. The thin optical plane in an opCMPAC, oriented perpendicular to the flow, strongly reduces the gas residence time in the sensor, thus minimizes gas transition broadening in the cell, drastically improving the TDLAS sensor response time. This enables much faster detection of dynamic concentration, pressure or temperature changes in the monitored flow. Further, the circular multipass absorption cell’s one-piece design makes the cell resistant to mechanical and thermal stress. Hence, the opCMPAC approach is ideal to be integrated in pipes/tube flows with only minimal disturbance of the cylindrical flow in the pipe [3,10,11,12].

As for all line-of-sight absorption measurements, in particular in open-path configurations, the effects of spatial heterogeneities in gas pressure, gas temperature or gas species concentrations has to be carefully considered and quantitatively evaluated with respect to systematic deviations or uncertainties caused by the interaction of the (possibly heterogenous) p, T, c-field with the spectral properties of the molecule, the particular chosen molecular transition and the fitting algorithm of the instrument [13,14,15,16,17,18]. The effects of temperature Inhomogeneities are particularly relevant due to their nonlinear effects on the intensity of the spectral line and hence the absorption line area and derived amount fraction [19]. Those effects have been investigated, e.g., for linear single-pass/two-section cell designs, but not for cylindrical flows and not with consideration for the characteristic “star polygon” beam pattern of CMPACs [16,19]. Such an investigation for circular cells has not been described in the literature and is needed for a high accuracy evaluation of dynamic concentration changes in cylindrical gas flows.

This work will investigate the effects of temperature and water vapor inhomogeneities measured with opCMPACs-based laser spectrometers (a) in the optimized *dynH_2_O* setup for dynamic hygrometer characterization at PTB [11,12], as well as (b) in strongly heterogeneous T fields generic for industrial process application, e.g., in pipe flows. Our measurements and simulations are focusing on the 7299.43 cm^−1^ H_2_O spectral line, frequently used for laser hygrometry, and cover the temperature range from 293 to 473 K at 1 atm of pressure as well as strong relative H_2_O concentration gradients of up to 60%. Using spectral simulations as well as spatially resolved gas temperature and H_2_O measurements in the *dynH_2_O* setup, we quantify the effect of T and H_2_O heterogeneity on the H_2_O concentration.

## 2. Materials and Methods

The investigated circular cell is integrated in the *dynH_2_O* setup as part of the SI traceable direct tunable diode laser absorption spectroscopy (dTDLAS) hygrometer used as a dynamic reference hygrometer in the setup, which aims to directly quantify generated, rapid H_2_O step changes of up to 10,000 ppm/s [12]. The essential background of the TDLAS [20,21,22] and dTDLAS methodology [23,24,25] and the properties of extractive CMPACs [5,6] have been described elsewhere. A short description of the relevant facts for the investigation of temperature and H_2_O amount fraction inhomogeneities is outlined below.

### 2.1. dTDLAS

In open-path direct tunable diode laser absorption spectroscopy, the concentration in amount fraction is derived by using the complete physical model, shown in Equation (1):

(1) $$c=-\frac{k_{B}\cdot T_{gas}}{S\left(T_{gas}\right)\cdot L\cdot p_{tot}}\underset{A_{line}}{\underbrace{\int^{\text{​}}ln\left(\frac{I\left(\nu \right)-E}{I_{0}\left(\nu \right)\cdot Tr}\right)\frac{d\nu }{dt}dt}}.$$

The model links the measured input quantities gas temperature $T_{gas}$, total gas pressure $p_{tot}$, detected laser intensity I behind the absorption path and initial laser intensity $I_{0}$ before the absorption path. In open-path configurations, background stray light could hit the detector, causing an emission “offset” E. Furthermore, absorbers such as dust particles can cause spectrally broadband (spectrally non-structured) transmission losses along the absorption path described by Tr. Instrument parameters to be considered are the optical absorption path length L and the dynamic, temporal tuning behavior dν/dt of the used distributed feedback (DFB) laser, which describes “how” the laser scans over the absorption line. Molecular parameters to be known are the integrated spectral line intensity *S*(*T_gas_*). S can be taken from line databases such as HITRAN or GEISA; in this work, however, we use more accurate data from our own measurements [26,27,28,29,30]. *k_B_*, finally, describes the Boltzmann constant. 

The spectrometer response without absorbers is described by the “baseline” which is composed of the broadband transmission losses Tr and the initial laser intensity $I_{0}$, fitted together using a third-order polynomial. The absorption line shape is approximated by a Voigt profile [31]. The integral term of Equation (1) describes the measured line area $A_{line}$. 

To decrease the degrees of freedom and stabilize the line fit, it is possible to pre-calculate the Lorentzian and Gaussian full width at half maximum of the Voigt profile from spectral line data and measured pressure and temperature, leaving only the line area, line position and polynomial coefficients of the baseline as free-fitting parameters. This process, as well as a detailed discussion and validation of Equation (1), is described in [3,23,25,32]. The link between the input parameters and the concentration via a full physical model makes the resulting concentration SI traceable—if all input parameters (L, $p_{tot}$, $T_{gas}$, $S\left(T_{gas}\right)$) are SI traceable—which eliminates the need to calibrate the instrument with a water vapor standard [32].

The need for a careful assessment of the effects of temperature inhomogeneities is illustrated by the nonlinear dependency of the spectral line intensity *S* with the temperature as shown in Equation (2):

(2) $$S\left(T\right)=S\left(T_{ref}\right)\frac{Q\left(T_{ref}\right)}{Q\left(T\right)}\frac{e^{\frac{-c_{2}E^{\prime \prime }}{T}}}{e^{\frac{-c_{2}E^{\prime \prime }}{T_{ref}}}}\frac{\left[1-e^{\frac{-c_{2}v_{ij}}{T}}\right]}{\left[1-e^{\frac{-c_{2}v_{ij}}{T_{ref}}}\right]}\;.\;$$

**Figure 1:**
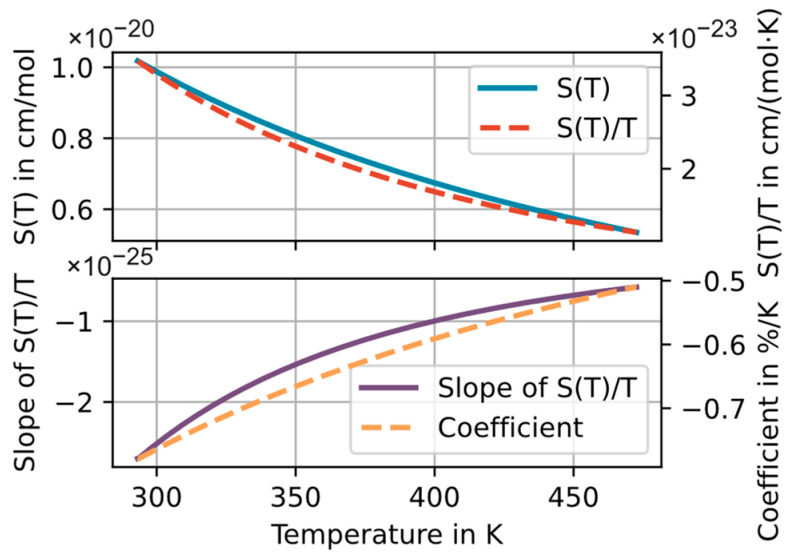
Temperature dependence of the spectral line strength *S*(*T*), the gas-density-corrected line strength S(T)/T and the first derivative of S(T)/T, as well as the relative temperature coefficient $\frac{\delta \;S\left(T\right)/T}{\;\delta \;T}/\left(S\left(T\right)/T\right)\cdot 100$ [16] in the temperature window between 293 and 473 K for the used H_2_O spectral line at 7299.43 cm^−1^.

The agreed reference temperature $T_{ref}$ in databases is 296 K. Q(T) denotes the partition sum, *E*″ the lower-state energy of the molecular transition, $v_{ij}$ the center wavenumber of the spectral line transition and $c_{2}$ the second radiation constant found in [26,27,33]. For the used H_2_O absorption line at 7299.43 cm^−1^, the temperature dependence of the line strength S(T) in the range from 293 to 473 K is shown in Figure 1. The temperature dependence of the determined amount fraction is also influenced by the gas density and hence better described by S(T)/T. Figure 1 (bottom) shows the temperature dependence of the local “sensitivity” (i.e., slope) of S(T)/T . This slope is a good indicator of the susceptibility of the spectral line to inaccuracies in the temperature measurement and/or temperature inhomogeneities along the optical path. The relative (percentage) temperature dependence of the determined amount fraction at a given temperature *T* is derived by normalizing the slope of S(T)/T by S(T)/T and multiplying the result by 100. This coefficient is shown on the bottom-right axis in Figure 1. The coefficient can be interpreted as the relative percentage change in the calculated concentration per Kelvin temperature error [16]. For the selected spectral line and the depicted temperature range, the coefficient ranges from −0.50%/K at 473 K to −0.78%/K at 293 K.

As an example, to suppress line strength and density temperature effects on the measured amount fraction at 293 K to a level of less than 1%, we would need a temperature accuracy (and homogeneity) of 2 K, which can be already quite demanding in industrial applications and with low-cost T-sensors.

The H_2_O spectral line used in this work is a frequently used [3,25,34,35] and well-studied [26,29,30] line. For high-accuracy concentration measurements, it is common to consider the effects of multiple surrounding lines by pre-calculating their contribution from given spectral data and subtracting their influence from the shape of the fitted main line. Because this work focuses on the effects of heterogeneities and not absolute concentration measurements, we simplify the mathematical “workload” in our spatially resolved simulations by concentrating on the temperature effects of the dominating main line at 7299.43 cm^−1^ only. For the given temperature and pressure conditions, the effect of this simplification has been calculated to be smaller than 1.1% with respect to the total concentration (calculated by simulating the effect of all H_2_^16^O and H_2_^18^O spectral lines documented in the HITRAN database between 7292.0 and 7308.0 cm^−1^ on the line area in the integration region from 7299.18 to 7299.68 cm^−1^).

### 2.2. Circular Multipass Absorption Cells—CMPAC

**Figure 2:**
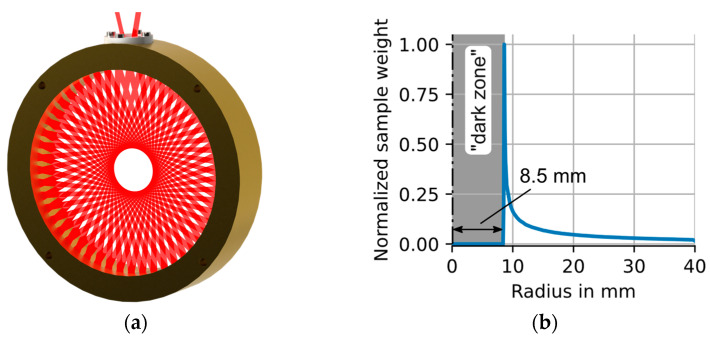
(**a**) CAD rendering of the CMPAC with laser beam. (**b**) Plot of the normalized sample weights of the beam path in our CMPAC configuration over the radius of the cell. The laser beam does not reach/measure in an inner circle of 8.5 mm radius. This we termed “dark zone”.

This work focuses on circular multipass absorption cells with planar beam patterns that can be described by two parameters only: the injection angle of the beam when entering the cell θ (the angle between the entrance and exit beam shown at the top of Figure 2a is 2θ) and the inner radius R of the cell. These two parameters define the number of reflection points including the entrance/exit point and hence the total absorption path length L in the CMPAC. The characteristic CMPAC beam pattern is referred to as a star polygon pattern [36]. 

The CMPAC beam pattern causes the laser to pass different regions of the cell cross section more often than others, effectively assigning different “weights” to different sections of the cell. These “weights” cause the same local gas sample or inhomogeneity to be recognized with different concentration values depending on the position of the sample in the cell. At the center of the cell, an area with the radius of R⋅sin(θ) is not interrogated by the laser beam, resulting in a “dark zone”. Here the local sample weight is zero. The fully circular (commercial) cell (not to be mixed with the “segmented” CMPAC [37]) used for the experiments and simulations has a diameter of 80 mm, an injection angle θ of 12.353° and 51 reflection points, resulting in a total path length of 3.986 m. 

Figure 2a shows a CAD rendering of the cell, and Figure 2b shows the normalized local sample weights of the cell averaged over the circumference at each radius r. The plot illustrates that the effects of spatial inhomogeneities in temperature or concentration will be amplified if they occur at the border of the “dark zone”. It should be noted that the problems caused by the “dark zone” and the sensitivity enhancement near the “dark zone” edge are reduced if the CMPAC is used for tubular flows, which result in radial, parabolic speed, concentration and temperature profiles, which consequently have lower relative spatial heterogeneities near the center of the tube than near the walls. 

A more detailed discussion of the geometric and optical properties of ring cells can be found here [5,6,36].

### 2.3. Experimental Setup

**Figure 3:**
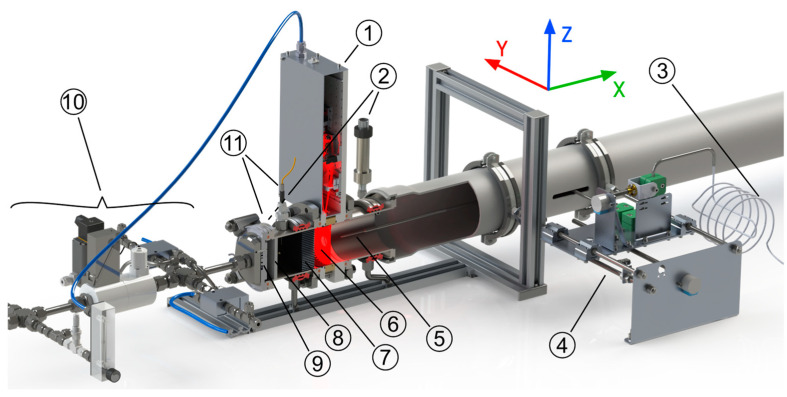
CAD rendering of the *dynH_2_O* setup with cut-through of the flow section, showing: ① the optics unit of the open-path reference dTDLAS hygrometer with the CMPAC, ② pressure sensors, ③ spatially scanned sampling line of the extractive SEALDH-II dTDLAS hygrometer, ④ automated positioning unit for the extractive gas probe, ⑤ gas extraction probe with critical orifice, ⑥ optical measurement plane, ⑦ aluminum honeycomb and ⑧ glass sinter filter to smooth the spatial flow profile, ⑨ injector plate, ⑩ base-flow gas mixing/switching/preparation unit and ⑪ stationary temperature sensors (fast thermocouple plus accurate platinum thermometer, PT100).

The investigated CMPAC is part of the open-path reference hygrometer of the *dynH_2_O* setup shown in Figure 3. The setup is designed to quantify the dynamic response behavior of small, point-sampling hygrometers (e.g., of the capacitive type) by generating well-defined, step-shaped H_2_O concentration variations (with minimized flow, temperature or pressure disturbance) and to record the response of the device under test (DUT), while simultaneously measuring the dynamics of the generated H_2_O concentration step with high temporal resolution and accuracy, and in particular without any gas sampling [11,12]. 

The *dynH_2_O* setup has been strictly optimized to minimize the temperature, flow and pressure changes that could occur during a concentration step, making it ideal to separately study the effects of concentration inhomogeneities in the optical measurement plane (Figure 3, ⑥) during a concentration step and the temperature distribution during constant concentration conditions. 

**Figure 4:**
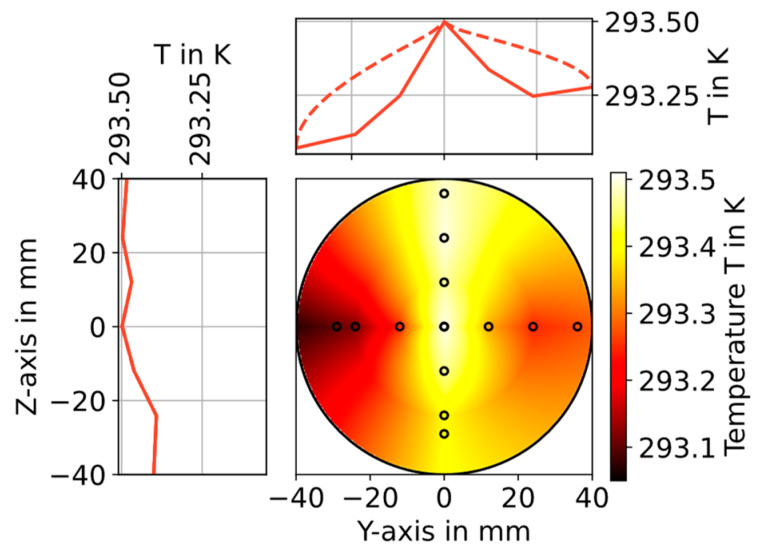
The 2D planar gas temperature distribution (color-coded) 14.5 cm behind the optical measurement plane of the circular cell derived from 13 local T-measurements (black circles) along the Y- and Z-axis. The plots on the top and left show the temperature profile along the Y- and Z-axis, respectively, as a solid line. The dashed line indicates the maximum value for each point on the Y-axis along the Z-axis and vice versa.

**Figure 5:**
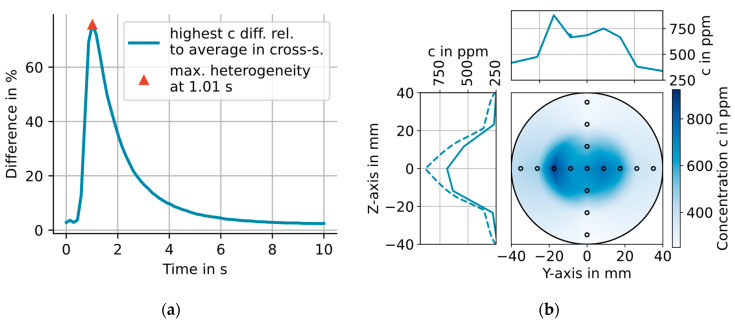
(**a**) Plot of the maximum relative concentration difference at one sample point in the cross section 7 cm behind the optical measurement plane relative to the average concentration in the cross section at that time. The maximum difference is reached at 1.01 s. (**b**) Concentration distribution in the cross section 7 cm behind the optical measurement plane of the circular cell, 1.01 s after the concentration step was triggered. The plots on the top and left in (**b**) show the concentration profile along the Z- and Y-axis, respectively, as a solid line. The dashed line indicates the maximum value for each point on the Y-axis along the Z-axis and vice versa.

To measure the spatial H_2_O distribution in the cross section, an extractive gas sampling probe with a critical orifice as inlet and an automated probe positioning unit were designed and integrated into the setup (Figure 3, ④ and ⑤). The probe is positioned 7 cm downstream of the optical measurement plane of the circular cell and can be traversed along the Y- and Z-axis as shown in Figure 3. 

The sample gas is additionally diluted with dry air directly behind the critical orifice in order to drastically reduce the residence time in the sampling line and to minimize adsorption in the probe. 

The sampled, diluted and pressure-reduced gas stream is traceably analyzed with the extensively validated and tested absolute dTDLAS hygrometer SEALDH-II [34,38], which was also used for numerous airborne campaigns [39]. The stationary, residual water vapor content in the dilution air is continuously monitored with a traceably calibrated dew point mirror (DPM). The average H_2_O concentration was found to be around 100 ppb.

The pressure in the gas cell of SEALDH-II is reduced to 120 mbar with a vacuum pump. This low pressure ensures that the orifice at the inlet of the probe is operating in a critical state, resulting in a constant sample gas flow of 0.5 standard liters per minute (=sl/min). The pressure drop right behind the inlet of the probe increases the volume of the sample gas by a factor of more than 8, and the gas sample volume is then further “increased” by a fixed, 5-fold dilution of the sample gas flow. These measures drastically reduce the residence time of the sample gas in the sampling system, significantly improving the response time of SEALDH-II by lowering the gas exchange time in the instrument to under 1 second and minimizing wall adsorption problems. The dilution air is controlled with a needle valve operating in its critical state. The resulting dilution ratio is calculated from the concentration values measured by the reference instrument of *dynH_2_O*, SEALDH-II and the DPM during a period with constant and homogeneous conditions in the flow section.

The temperature distribution is measured with a traceably calibrated PT100 with a precision of ±0.05 K. The accuracy of the PT 100 in this configuration is estimated to be better than ±0.3 K. The sensor is manually positioned at several points along the Y- and Z-axis in a cross section of the setup 14.5 cm behind the optical measurement plane of the circular cell (small circles in Figure 4).

## 3. D-Temperature and H_2_O Concentration Distribution Measurements

### 3.1. Temperature Measurements

The spatial profile of the gas temperature was measured at seven points along the Y- and Z-axis, with the tube center point included in both measurement series. The effects of concentration steps on the temperature distribution in the cross section were investigated using a thin-wire thermocouple (type T, 0.5 mm diameter) for the detection of fast temperature fluctuations. The investigation showed no detectable influence (detection limit 3 × standard deviations is 0.1 K) of the generated H_2_O concentration steps from 300 to 3300 ppm (at 1005 mbar) on the temperature in the flow section, allowing the investigation of the temperature distribution in stationary humidity conditions and with a more accurate PT100 temperature sensor. Each point shown as small circle in Figure 4 was measured for 20 min using the PT100. The temperature during the last two minutes was averaged and room temperature influences were compensated for. The precision of the used PT100 is 0.05 K, and the average standard deviation during the evaluated two minutes is 0.01 K. The measured datapoints are linearly interpolated along the Y- and Z-axis and along the circumference to get the approximation of the 2D temperature distribution shown in Figure 4.

The average temperature In the full (interpolated) temperature field shown in Figure 4 is 293.33 K with a standard deviation of 0.092 K (or 0.03% relative). The gas flow at the center is 0.43 K warmer than the gas at the left wall, and the warm gas rises to the top of the flow section as expected.

### 3.2. 2D H_2_O Concentration Measurements

With the setup described in Section 2.3, the dynamic H_2_O concentration was measured during 15 (300 to 3300 ppm) concentration steps at the 15 locations shown as small circles in Figure 5b. The excellent repeatability of the generated H_2_O steps enables the synchronization of the concentration steps based on the recorded trigger times of the valves [12]. The concentration distribution in the CMPAC cross section is calculated analog to the temperature distribution, for every point on the time axis starting at the generation of the step and ending 10 s later. The difference between the H_2_O average and the highest H_2_O concentration in each calculated distribution relative to the average concentration in the cross section at that time is shown in Figure 5a. The highest relative difference of 75.7% is reached 1.01 s after the step was triggered. 

This dynamic spatial heterogeneity is caused by the radial dependence of the flow speed, being highest in the tube center (as expected by the Hagen–Poiseuille law). The H_2_O front therefore arrives first in the tube center and last at the walls in the CMPAC, causing this dynamic spatial H_2_O heterogeneity. The concentration distribution at the time of the largest heterogeneity is shown in Figure 5b. The spatial average of the H_2_O concentration over the full cross section is 501.7 ppm with a standard deviation of 197.2 ppm (or 39.3% relative). The concentration distribution shown in Figure 5b will be used as (worst-case) input for the simulation described in the following section.

## 4. Simulating the Effects of Temperature and H_2_O Concentration Inhomogeneities on the Line-of-Sight Averaged Concentration Measured with the CMPAC

### 4.1. Simulating the Effects of the Measured Spatial Gas-T and H_2_O Distributions

**Figure 6:**
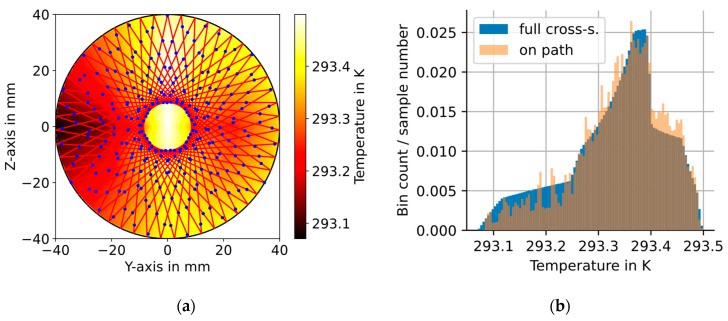
(**a**) The 2D planar temperature distribution (color-coded) in the CMPAC, as described in Section 3.1, with the laser beam pattern (red lines), and the local sample points along the optical path (blue dots, only every 100th sample point is shown) superimposed. (**b**) Histogram of the local temperatures along the optical path (orange) compared to the local temperatures in the entire cross section (blue) derived from the interpolated gas temperature measurements (see Figure 4).

To simulate the effects of temperature and concentration inhomogeneities in the measurement plane of the CMPAC on the resulting line-of-sight averaged H_2_O concentration value, the temperature/concentration distribution along the optical path needs to be extracted from the 2D distributions described in Section 3. This is performed by transferring the interpolated distributions on an 800 × 800 grid, each cell with a 0.1 × 0.1 mm size. The values in the grid are sampled along the optical path in equidistant steps every $\sqrt{2}\times 0.1$ mm to avoid two consecutive samples to be drawn from the same cell. The resulting sample pattern is illustrated in Figure 6a, where every 100th sample point along the optical laser path is shown as a blue dot. 

The histogram in Figure 6b shows the gas temperature distribution for the samples (a) along the optical path (orange) and (b) as the distribution in the entire cross section (blue). As expected from the sample weights, shown in Figure 2b, the samples drawn along the optical path slightly overrepresent the higher temperatures that are found near the center of the pipe, while the lower temperatures, which are found near the walls of the pipe, are slightly underrepresented. The concentration distribution along the optical path is determined in the same way. 

The local, discrete absorbance simulation along the line of sight calculates the absorbance spectrum of the 7299.43 cm^−1^ H_2_O line between 7292.0 and 7308.0 cm^−1^ with a spectral step size of 0.002 cm^−1^ and determines the H_2_O line area by numerical spectral integration between 7299.18 and 7299.68 cm^−1^ (±0.25 cm^−1^ around the center of the main line). The absorbance is calculated (a) as the sum of the absorption during 1 mm long steps on the optical path, with the local gas temperature, H_2_O concentration and spatially homogenous gas pressure and (b) for the total length of the optical path (3.96 m) assuming perfect homogeneity, i.e., with just one temperature, concentration and pressure value. Equation (1) shows that the difference in the line area resulting from the two approaches will be proportional to the difference in the calculated concentration value. The line area calculated as the sum of steps along the optical path can be interpreted as the “true” value taking into account all heterogeneities. This, hence, can be used to study the effects of different, “simplifying” assumptions, commonly conducted under “real-world” field conditions, such as, e.g., to assume a single (measured) temperature and concentration value would represent the situation in the entire optical path. This method is developed further from [16]. The effects of temperature, concentration and pressure inhomogeneities within *dynH_2_O* are orthogonal and hence independent, enabling the separated study of their influences by considering changes in one parameter while assuming spatially homogenous conditions for the others. This is conducted for the three most relevant scenarios for *dynH_2_O* for a fixed total pressure of 1 atm (1013.25 mbar). 

The real-world scenarios compared below differ in the quality of the assumptions made to calculate the single temperature or concentration value to determine the line area, which is compared to the line area obtained by numerical integration over the measured and interpolated temperature/concentration distribution along the optical path in the CMPAC. The scenarios we compare are as follows:

1. Using the average temperature/concentration on the optical path. The deviations found in this scenario can be seen as the pure “spectroscopic effects”, e.g., from the nonlinear temperature dependence of the line intensity discussed in Section 2.1.
2. Using an average temperature/concentration calculated for the entire cross section.
3. Using a single temperature/concentration at the center of the ring cell. This scenario is especially relevant for practical applications of the circular cell where the temperature is often measured with a single temperature sensor at the center.

Scenario 1 and 2, however, would cause a significant (unrealistic) amount of effort/time to determine in a real-world application. These scenarios hence serve as hypothetical, but technically less realistic reference cases.

Table 1 shows the results of the simulations for our technically near optimal *dynH_2_O* case. The inhomogeneities in the concentration distribution described by the relative standard deviation in the cross section as shown in Section 3 are more than 1300 times larger than the inhomogeneities in the measured temperature distribution. The resulting effects of the temperature inhomogeneities are therefore more than three orders of magnitude smaller than the effects of the concentration inhomogeneities across all scenarios shown in Table 1. The largest difference of 29.0% occurs when comparing the concentration at the center of the cell with the simulated measured concentration. This result illustrates the importance of additional post-processing steps as described in [11,12] before comparing the concentration determined with the CMPAC to the values from a DUT with a small active area or with an extractive probe at the center of the flow section in dynamic conditions. 

The deviation of −16.1% between scenario 1 (average on the optical path) and scenario 2 (full cross section average concentration) clearly shows that the common assumption that the concentration that is measured approximates the average in the cell is not (!) valid for large concentration inhomogeneities. 

**Figure 7:**
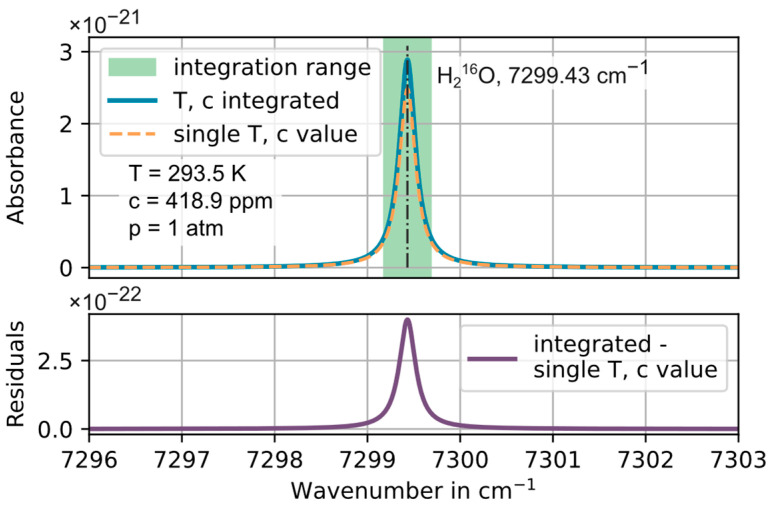
**Top**: Simulated, integrated absorbance spectrum of the 7299.43 cm^−1^ H_2_O line along the CMPAC light path, taking into account in scenario (a) (in blue) the measured and interpolated spatial heterogeneities in gas temperature and H_2_O concentration and in scenario (b) (dashed orange) using the average concentration in the cross section and the temperature at the ring center as single “average” values for the entire optical path. The range in which the line area is determined by numeric integration is shown in green. **Bottom**: Residual between scenario (a) and (b). The relative difference in the peak absorption at the line peak between scenario (a) and (b) is 13.4%.

Figure 7 compares the simulated H_2_O spectra with the temperature at the center and the average concentration in the cross section used for the calculation (combination of scenario 2 and 3). Here the relative deviation of the peak absorption reaches 13.4%.

### 4.2. Simulating the Effects of Severe T Inhomogeneities at Center Temperatures of up to 473 K

In technical gas flows at elevated temperatures above room temperature, the temperature heterogeneity can be expected to be much larger than in the almost ideal *dynH_2_O* case with only 0.03% relative standard deviation in T. Hence it does make sense to quantify the influence of larger boundary layer profiles in the gas temperature using the simulation approach described before. To simulate a realistic technical situation, we assume a hot gas flow in a tube with constant wall temperature of 293 K (20 °C), while the gas flow has a core temperature from 293 to 473 K (200 °C), forming a parabolic boundary layer with a thickness of 41% of the tube radius. This generic situation covers a broad range of typical industry scenarios.

We also determine the effect of four different “cost vs quality” choices of temperature information. Case (D) has the lowest cost: we do not invest in a gas-T measurement and just use the wall temperature instead. In Case (C) we invest in a single gas-T probe and measure the core gas temperature (but “ignore” the T boundary layer). In Case (B) we use the average temperature of the full flow cross section. This option is technically already quite challenging and costly to achieve and hence will not be found in industry. In Case (A) the ideal T information would be the real temperature average along the optical path, which is technically really difficult to access [40] and hence serves as an “ideal reference” case. 

To evaluate these scenarios and quantify the systematic errors in H_2_O caused by the thermal boundary layer and the choice of temperature information, we repeated the simulation with 40 different parabolic temperature profiles, with constant pipe wall temperatures and increasing core gas temperatures of up to 473 K. The water vapor concentration is assumed to be homogeneous with 1000 ppm in all simulations. The gas pressure was fixed to 1 atm.

**Figure 8:**
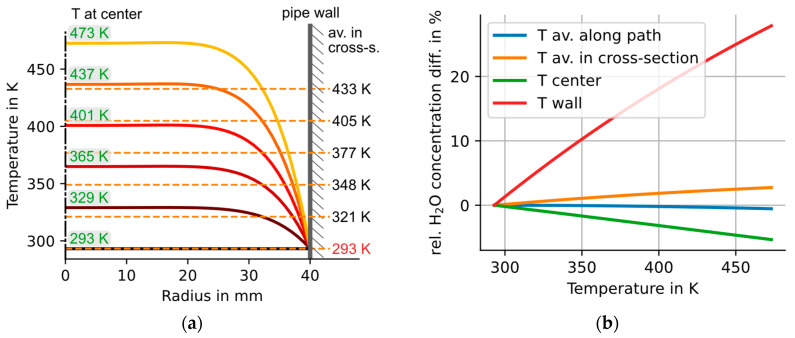
(**a**) Set of six temperature profiles used to calculate the results shown in (**b**), with the temperature at the tube center shown on top of the profiles on the left and the average temperature across the full T profile shown as dashed line. (**b**) Relative difference between the line area calculated by integrating the temperature distributions shown in (**a**) along the optical path to the line area calculated with a single temperature value. The single temperature values used to evaluate the simulated absorption spectrum (see Figure 7) are (1) the average temperature along the optical path, (2) the average temperature in the full 2D cross section, (3) the temperature at the tube center and (4) the wall temperature (which was in the simulations fixed to 293 K). The pressure and concentration distributions for all those scenarios were identical and assumed to be homogeneous: p = 1 atm and H_2_O = 1000 ppm.

The temperature profiles with the lowest and highest center temperatures, as well as four profiles in between, are shown in Figure 8a. The temperature profile with the highest center temperature (473 K) has the largest relative standard deviation (1σ) in temperature of 31.5% over the full 2D cross section. 

The relative differences between the ideal line area, calculated with consideration of the full temperature profile, and the line area calculated with a simplified, single temperature value are plotted over the core gas temperature of each profile in Figure 8b. 

The simulation shows (Figure 8b) that the systematic error in H_2_O rises proportional to boundary layer “delta-T”, i.e., the maximum temperature difference across the thermal boundary layer. The H_2_O error also correlates strongly with the quality of the temperature information: The lowest-cost approach (D, ignoring core gas-T) causes the largest relative H_2_O differences of up to 27.8%. Case C (a single T sensor for the core-T) reduces the error magnitude by over a factor of five, to −5.3%. Case B (area averaged gas-T) still leads to deviations of up to +2.5%, while the path-averaged gas temperature (Case A) yields the best results, with sub-percent deviations to the ideal line-of-sight integrated result.

## 5. Discussion of Results

The temperature and water vapor concentration inhomogeneities in the optical circular cell of the *dynH_2_O* setup were measured. The average temperature in the cross section of the CMPAC was found to be 293.3 K at an average H_2_O concentration of 501.7 ppm. The inhomogeneities expressed as relative standard deviations in the cross section are 0.03% for the temperature and 39.3% for the H_2_O concentration, indicating an excellent T homogeneity and a strong H_2_O boundary layer profile. 

A numerical, spectroscopic simulation was used to investigate the effects of the 2D temperature and H_2_O distribution on the H_2_O concentration calculated using the line area of the 7299.43 cm^−1^ H_2_O spectral line. The results were compared to the H_2_O concentration calculated with three different assumptions for the temperature and concentration values: (1) average value along the optical path, (2) average value in the entire cross section and (3) the value at the center of the tube. 

For the temperature distribution in *dynH_2_O* the resulting relative differences in all three scenarios are smaller than 0.01%. The small temperature inhomogeneities in *dynH_2_O* have therefore a negligible influence on the retrieved H_2_O concentration. This shows that the measures taken to homogenize the temperature in *dynH_2_O* (described in [11,12]) are effective and sufficient. 

The effects of the concentration inhomogeneities stay below 0.02% when compared to the average concentration along the optical path. However, they reach −16.1% when compared to the average concentration in the cross section and +29.0% when compared to the concentration at the center of the gas cell. The large deviation to the average concentration in the cross section can be linked to the characteristic beam pattern of the circular cell, which leads to a “local sampling bias”. H_2_O close to the mirror surface of the cell is undersampled due to the smaller laser beam density. The “dark zone” at the center is not sampled at all, while values outside, near the edge of the “dark zone” are oversampled and hence overweighted. 

A typical use case for *dynH_2_O* is the characterization of the dynamic response behavior of a small diameter chip-hygrometer or a single tube gas sampling system which is placed in the core of the flow section of the setup. To compare the results of these small-sized DUTs with the measurements from the spatially integrating TDLAS reference instrument, we use a simulation to determine and remove the effects of the concentration inhomogeneities and sampling biases. The investigation described in this work illustrates the importance of such additional steps in order to compare a measurement at the center with a measurement obtained by using the CMPAC in dynamic conditions [12].

The effects of strong temperature boundary layer inhomogeneities are investigated by simulating the effects of parabolic temperature profiles with a fixed wall temperature of 293 K and increasing core temperatures from 293 to 473 K. For the largest investigated wall-to-center temperature difference of 180 K, our simulations predict relative deviations in the calculated concentration of (a) 27.8% when the wall temperature is used for the evaluation—instead of the temperature distribution along the optical path—and (b) deviations of −5.3% when the center temperature is used for the evaluation. 

This comparison clearly illustrates the importance of a more detailed investigation of the effects of temperature inhomogeneities in cylindrical pipe flows, especially if large temperature “gradients” are present or higher accuracy concentration measurements are needed. 

## 6. Conclusions

The effects of inhomogeneities in the gas temperature or H_2_O concentration distribution in CMPACs on the concentration determined with line-of-sight absorption spectroscopy were investigated. The characteristic “star polygon” beam pattern in a CMPAC causes different regions inside the optical plane to affect the resulting measurement more than others, effectively assigning variable, local “sample weights”, equivalent to the local laser beam density in this region. We were able to show that this local weighting of the CMPAC beam pattern can lead to strong systematic deviations in the path-averaged target gas concentration. This is in particular the case when the target gas is unevenly distributed in the CMPAC sampling plane. This systematic deviation can be as large as −16.1% in the measured case of the strong H_2_O boundary layer of the *dynH_2_O* setup. The common assumption that the derived concentration represents the area-average concentration in the cell therefore does not hold true for large concentration inhomogeneities and needs to be carefully taken into account, particularly in open-path CMPAC applications in flows with strong boundary layers.

If the gas temperature in the CMPAC plane is not homogenous, the path-averaged concentration value determined with a CMPAC is dependent on (a) the shape and magnitude of the T-heterogeneity, and (b) the choice for a “representative” temperature used for the spectroscopic evaluation. Here a potential strong nonlinear influence of the gas temperature on the particular spectral line intensity (in our case at 7299.43 cm^−1^) needs to be considered. This can result in strong systematic deviations in the “spectroscopic” concentration from the “true” concentration. In heterogeneous temperature conditions this can even be the case if the average temperature along the optical path is used for the evaluation. Our simulation of the effects of different, commonly used temperature measurement ”choices” on the accuracy of the resulting concentration value additionally showed that the influence of the “spectroscopic effects” can even be surpassed by an inadequate placement of temperature sensors. For example, if—for the case of a strong thermal boundary layer with a delta-T of 180 K—a single temperature sensor is used to measure T_max_ at the hot cell center, then this would lead the described laser hygrometer to a relative deviation of −5.3% between the “true” and the calculated concentration. Thus, we stress the importance of accurate and representative temperature information as well as sufficient knowledge of the shape and magnitude of T heterogeneities. In the end, it is not only the accuracy, but also the choice, quality, number of T-sensors and their placement in the heterogenous gas flow which critically influence the accuracy of an open-path concentration measurement and the magnitude of the potentially severe systematic deviations.

## Figures and Tables

**Table 1:** Results of the simulation describing the difference in percent between the line area calculated by integrating the temperature/concentration along the optical path and using a single value defined by one of the three scenarios for the calculation.

	Scenario 1 Av. Along Path	Scenario 2 Av. in Cross Section	Scenario 3 Center
**Temperature**	0.00000161%	0.000544%	−0.00838%
**Concentration**	0.0146%	−16.1%	29.0%

## Data Availability

The data are available from the authors upon request.
